# Human Umbilical Cord Wharton Jelly Cells Treatment Prevents Osteoporosis Induced by D-Galactose

**DOI:** 10.1155/2022/4593443

**Published:** 2022-07-20

**Authors:** Qiang Wang, Zhiqiang Gao, Kai Guo, Jiawei Lu, Feng Wang, Tongde Wu, Yufeng Huang, Desheng Wu

**Affiliations:** Department of Spine Surgery, Shanghai East Hospital, Tongji University School of Medicine, 150 Jimo Rd., Shanghai 200120, China

## Abstract

**Methods:**

Sixteen male mice were randomly divided into 4 groups: control (ordinary feeding), D-gal (D-galactose) group, D-gal + MSC (human umbilical cord Wharton jelly cells), and D-gal + MSC-TNF*α* groups. Except for the control group (fed with same amount of saline solution), other mice received gastric feeding of 250 mg/kg D-galactose every day for 8 weeks. TNF*α* (10 ng/mL for 24 h) cocultured or noncocultured HUCWJCs (5 × 10^5^) were suspended in 0.1 ml of sterile PBS and injected into tail veins every other week in D-gal + MSC-TNF*α* and D-gal + MSC groups, respectively, and only 0.1 ml of sterile PBS for control and D-gal groups. The bone mass was detected by qPCR, ELISA, microcomputed tomography (*μ*CT), and hematoxylin-eosin staining. Proliferation, apoptosis, and differentiation of periosteal-derived osteoblasts (POB) were assessed. Transwell assay and scratch healing were performed to detect POB migration and invasion ability. The effect of HUCWJCs on POB signaling pathway expression was evaluated by immunoblotting.

**Results:**

The malondialdehyde (MDA) in serum was higher and superoxide dismutase (SOD) was lower in the D-gal group compared to the other groups (*p* < 0.05). Mice in D-gal group showed significantly decreased bone mass when compared to the control group, while HUCWJCs treatment partially rescued the phenotype, as demonstrated by *μ*CT and histology (*p* < 0.05). Mechanically, HUCWJCs treatment partially promoted proliferation and migration and decreased apoptosis of POB induced by oxidative stress via activating the mitogen-activated protein kinase (MAPK) signaling pathway.

**Conclusion:**

HUCWJCs can prevent the progression of osteoporosis by inhibiting oxidative stress, which may act by regulating osteoblasts fate through the MAPK signaling pathway.

## 1. Introduction

Osteoporosis is a common disease characterized by decreased bone density and increased risk of fracture that affects hundreds of millions of people worldwide [1]. Many studies suggested that the increased oxidative stress level is closely related to the occurrence of osteoporosis [2–4]. Oxidative stress has a key role in transmitting intracellular signals that regulate proliferation, differentiation, repair process, and inflammation of cells [5]. It was suggested that oxidative stress inhibits bone formation [6]. In addition, decreased glutathione levels and defensive antioxidant capacity have been found in elderly patients with osteoporosis and secondary osteoporosis caused by chronic intestinal disease (IBD) [7, 8]. The role of D-galactose in inducing oxidative stress has been widely used to establish animal model of premature aging and the aging process induced by D-galactose is like the natural aging process [9–11]. Human umbilical cord Wharton jelly cells (HUCWJCs) have been used to treat various inflammatory diseases due to their excellent anti-inflammatory properties. Previous studies have shown that the decrease of resident mesenchymal stem cells is closely related to the occurrence of osteoporosis. Exosomes from human adipose-derived mesenchymal stem cells have been often used for bone tissue engineering and repair of bone defects [12]. These exosomes ameliorate inflammation and promote alkaline phosphatase (ALP) activity, calcium deposition, osteocalcin (OCN) secretion, and proliferation and inhibit apoptosis in osteoblasts [13]. Ge et al. suggested that these exosomes are sufficient to reverse the symptoms of osteoporosis in mice [14].

So far, there is no literature to report whether the D-galactose-induced osteoporosis mouse model can be treated by HUCWJCs. We hypothesized HUCWJCs could be a mediator in osteoporosis therapy and pro-inflammatory factors (TNF*α*) may inhibit its anti-inflammatory effects. To address the question, we characterized oxidative stress in mice, after which the bone mass of lower limbs was quantified and the signaling pathway expression was explored.

## 2. Materials and Methods

### 2.1. Animals

Since estrogen can prevent the development of osteoporosis and its anti-inflammatory effect interferes with the establishment of D-galactose induced osteoporosis model, male mice are more suitable for this study [15, 16]. Accordingly, sixteen wild-type male C57BL/6J mice were purchased from Shanghai SLAC Laboratory Animal Company Limited. All the animals were housed in an environment with a temperature of 22 ± 1°C, a relative humidity of 50 ± 1%, and a light/dark cycle of 12/12 hr and were given water and food ad libitum. The dose of D-galactose inducing oxidative senescence in mice (200 mg/kg/d for eight weeks) was taken for reference [17, 18]. Human umbilical cord Wharton jelly cells (HUCWJCs) can inhibit the occurrence of osteoporosis by inhibiting expression of TNF*α* and also exhibit good antioxidant effect by inhibiting TNF*α* related signaling pathways [19]. However, it was also suggested that TNF*α* promotes osteogenic differentiation of HUCWJCs [20]. To explore whether TNF*α* inhibits the anti-inflammatory effect of HUCWJCs and intervenes the effect of HUCWJCs on osteoporosis, in our study, mice were randomly divided into 4 groups: control (ordinary feeding), D-gal (D-galactose) group, D-gal + MSC (human umbilical cord Wharton jelly cells), and D-gal + MSC-TNF*α* groups. All mice were fed conventional diet. At 6 months of age, except for control group, all mice received gastric feeding of 250 mg/kg D-galactose every day for 8 weeks. At the same time, TNF*α* (10 ng/mL for 24 h) cocultured or noncocultured HUCWJCs (5 × 10^5^) were suspended in 0.1 ml of sterile PBS and injected into tail veins every other week in D-gal + MSC-TNF*α* and D-gal + MSC groups, respectively, and only 0.1 ml of sterile PBS injection for control and D-gal groups. At 8 months of age, blood was collected and analyzed by ELISA (Jiangsu Yutong Biological Technology Company). Mice were then euthanized using pentobarbital (100 mg/kg, IP injection), and the lower extremities of the sacrificed mice were dissected and immediately put in 4% paraformaldehyde for 24 h and then transferred to phosphate-buffered saline.

### 2.2. Isolation and Culture of Cells

Umbilical cords from full-term deliveries were obtained from Shanghai East Hospital of Medical College of Tongji University after the written informed consent of mothers. The study ethics and trial protocol have been approved by the Human Cell Clinical Research Ethics Committee of Shanghai East Hospital, Tongji University School of Medicine, and Ethics Committee on Stem Cell Clinical Research. The experiments were performed in accordance with the requirements of the local ethical committee. Arteries and vessels were removed from the umbilical cord. Wharton's jelly was split into pieces and cultured in DMEM/F12 supplemented with 1% nonessential amino acids, two mmol/L glutamine, 10% heat-inactivated FBS, 100 U/mL penicillin, and 100 g/L streptomycin in an incubator at 37°C with 5% CO_2_. One week later, a cell clone of HUCWJCs could be observed around Wharton's jelly slices. HUCWJCs were cocultured with TNF*α* (10 ng/mL R&D Systems) for 24 h before infusing into a caudal vein, which was referred to relevant literature [21, 22]. Periosteal-derived osteoblasts (POB) were provided by arthrosis colleagues. POB pretreated with or without H_2_O_2_ (400 uM) for 3 h were ROS and control group, respectively, while POB pretreated with H_2_O_2_ and followed by culturing with HUCWJCs or HUCWJCs-TNF*α* for 48 h were ROS + MSC group and ROS + MSC-TNF*α* group, respectively. This grouping method is applicable to the subsequent grouping of experimental steps, including the EDU, TUNEL, mineralization assay, migration, invasion, and signaling pathway expression examination.

### 2.3. Micro-CT

The right lower extremities were harvested from the mice following euthanasia. Samples were fixed in 4% paraformaldehyde for 24 h and then scanned using a micro-CT system (MicroCT 40 and vivaCT; ScancoMedical, Bassersdorf, Switzerland). Global thresholding procedure was used to separate the calcified tissues from soft tissues. The distal epiphyseal of femur was scanned at 8 *μ*m resolution to analyze trabecular bone mass. The following parameters were determined for the trabecular bone at the distal femur: bone mineral density (BMD), relative trabecular bone volume (BV/TV), trabecular spacing (Tb. Sp), trabecular number (Tb. N), and trabecular surface area/bone volume (Tb.BS/BV). Three individuals, all of whom were blinded to the grouping, performed the radiographic analysis.

### 2.4. Histology and Mineralization Assay

After *μ*CT test, the right lower extremities were treated with 0.25 M ethylenediaminetetraacetic acid (EDTA) for decalcification for 3 weeks. The samples were then embedded in paraffin, and 6 *μ*m sections were prepared using a standard microtome (RM2255, Leica). Sections were stained with hematoxylin-eosin (H&E) (SD0146, Shanghai Si Ding Biological Technology Company). For H&E staining, trabecular bone area/total area was calculated with Image-Pro Plus V6.0.0. For mineralization assay, four groups of cells were treated under different conditions (as described above), then followed by culturing with osteogenic differentiation medium including *α*MEM supplemented with 10% fetal bovine serum (FBS), 1% streptomycin (100 mg/mL), penicillin (100 u/mL), 100 nM dexamethasone (Sigma), and 50ug/mL ascorbic acid (Sigma) as well as 10 mM *β*-glycerophosphate sodium (Sigma). Consequently, cells were subjected to Alizarin Red S staining at day 14 (60504ES25, YEASEN, China) to identify calcium deposits in the cell layers of osteoblasts. Images were acquired using a microscope (Olympus, Tokyo, Japan). For quantitative analysis, cells were destained with ethyl pyridine chloride for 30 min at room temperature and transferred to a 96-well plate to measure the absorbance at 550 nm using a microplate reader.

### 2.5. Real-Time RT-PCR Analysis

The left lower extremities from 8-month-old mice were dissected. The samples were subsequently placed in Trizol (15596026, Thermo Fisher Scientific, USA). Total RNA was then extracted according to the manufacturer's instructions, and cDNA was synthesized from 1 *μ*g of total RNA. qPCR was performed with SYBR Green PCR Master Mix (Q711-02, Vazyme, USA). Thermocycler program consisted of an initial hot start cycle at 95 °C for 3 min, followed by 40 cycles at 95 °C for 5 sec and 60 °C for 34 sec. All qPCR reactions were run in triplicate and normalized to the expression of GAPDH. The calculation of the relative expression was performed according to the 2-ddCT method. Each reaction was run in triplicate and independently repeated 3 times. The sequences and product lengths for each primer pair were as follows: ALP (Forward: 5′-AGTCAGGTTGTTCCGATTCA-3′;Reverse:5′-GGGCATTGTGACTACCACTC-3′);OCN (Forward:5′-AGCAGGAGGGCAATAAGGT-3′; Reverse:5′-ACTTGCAGGGCAGAGAGAGA-3′); OPN (osteopontin) (Forward:5′-ACCATGAGATTGGCAGTGAT-3′; Reverse:5′-GTTGGGGACATCGACTGTAG-3′); GAPDH (Forward:5′-CACATTGGGGGTAGGAACAC-3′; Reverse:5′-AACTTTGGCATTGTGGAAGG− 3′).

### 2.6. TUNEL Assay

Terminal deoxynucleotidyl transferase-mediated dUTP-biotin Nick End Labeling (TUNEL, Beyotime, Beijing, China) was used to visualize cell apoptosis. POB were suspended in a serum-free medium and plated in the upper chamber at a density of 4 × 10^4^ cells/dish and the four groups of POB were treated under different conditions. Apoptotic cells were then counted under 5 visual fields with FLUOVIEW software. TUNEL staining was performed by three researchers who were blinded to the grouping.

### 2.7. Transwell Assay and Scratch-Wound Assay

For analysis of invasion capability, POB were suspended in 250 *μ*L serum-free DMEM/F12 and added into the upper chamber of a Transwell plate (Corning, New York, NY, USA) with an 8 *μ*m pore size polycarbonate filter. After complete culture medium with 10%FBS with or without H_2_O_2_ (400 uM for 3 h), the medium was changed into a 600 *μ*L complete culture medium with 10% FBS + HUCWJCs/HUWJCs-TNF*α* in the lower chamber. After 48 hours, cells in the lower chamber were stained by crystal violet, and a cotton swab was used to remove cells on the upper surface of the filter and observed under an inverted microscope. For scratch-wound assay, after the cell reached 90% confluence, a line was drawn using a marker on the bottom of the dish, and then a sterile 1,000 *μ*l pipet tip was used to scratch three separate wounds through the cells, moving perpendicular to the line. The cells were gently rinsed twice with PBS to remove floating cells and incubated in 5 ml of medium containing 10% FBS in 37°C, 5% CO2/95% air environment. Images of the scratches were taken by using an inverted microscope after 0.12 and 36 h of incubation and quantified by calculating the wound healing area of cells in the scratched area.

### 2.8. Statistical Analysis

Statistical analysis of the data was performed by *t*-tests or one-way analysis of variance (ANOVA). All data are presented as means ± SD. *p* < 0.05 was considered as statistical significance. Each experiment was performed in triplicate.

## 3. Results

### 3.1. HUCWJCs Partially Rescue the Osteoporosis Phenotype Induced by Oxidative Stress

**Figure 1:**
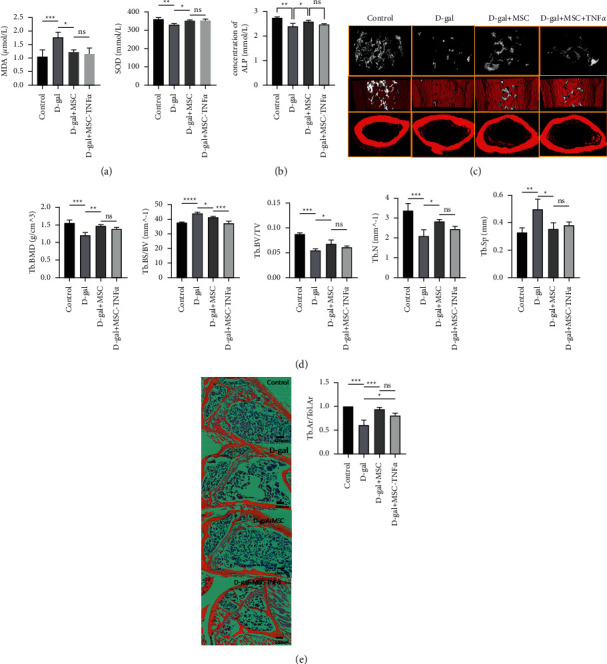
HUCWJCs partially rescue the osteoporosis phenotype induced by oxidative stress. (a) ELISA result of malondialdehyde (MDA) and superoxide dismutase (SOD) in serum. (b) ELISA result of alkaline phosphatase (ALP) in serum. (c) Representative *μ*CT scans of the distal femur showing the 3D reconstructed trabecular bones of mice at 8 months of age and quantitative analysis of the trabecular bone mass density (Tb.BMD), trabecular bone surface area/tissue volume (Tb.BS/BV), percentage of bone volume (BV/TV), trabecular thickness (Tb. Th), trabecular number (Tb. N), and trabecular spacing (Tb. Sp) (*n* = 3). (d) Quantification of the *μ*CT result. (e) Hematoxylin & eosin staining of 8-month-old mice showed the trabecular bone at the distal femur and quantification of trabecular bone tissue area/total area. Scale bar, 200uM. All data is reported as the mean ± SD. Statistical significance was determined by one-way ANOVA. ^*∗*^*p* < 0.05_,_^*∗∗*^*p* < 0.01, ^*∗∗∗*^*p* < 0.001; ns = not statistically significant.

To investigate the effect of HUCWJCs on osteoporosis in mice, we established the osteoporosis model by D-galactose induction. After 8 weeks of feeding with 250 mg/kg D-galactose and HUCWJCs treatment, malondialdehyde (MDA) in serum in D-gal group (1.768 ± 0.185umol/L) and in D-gal + MSC group (1.220 ± 0.091 umol/L) was both higher than that in control group (0.442 ± 0.261 umol/L) (all *p* < 0.05). Moreover, superoxide dismutase (SOD) in D-gal group (331.033 ± 4.535 umol/L) was lower than that in control group (359.887 ± 9.103 umol/L) (*p* < 0.05), while there was no significant statistical difference between D-gal + MSC group (351.620 ± 5.021 umol/L) and control group (*p* > 0.05), which means that the inflammation level of mice was increased and the antioxidant capacity was downregulated after D-galactose induction (Figure 1(a)). Alkaline phosphatase (ALP) in serum in D-gal group (2.398 ± 0.116 mmol/L) and D-gal + MSC group (2.595 ± 0.048 mmol/L) was also lower compared to the control group (2.735 ± 0.435 mmol/L) (*p* < 0.05); In addition, parameter of ALP in D-gal + MSC group was significantly higher than the D-gal group (*p* < 0.05). There is no statistical significance between D-gal + MSC and D-gal + MSC-TNF*α* groups (Figure 1(b)). *μ*CT showed the lowest bone mass at the distal femur in the D-gal group after treatment for 8 weeks. Parameters of trabecular bone mineral density (Tb.BMD), trabecular surface area/bone volume (Tb.BS/BV), relative trabecular bone volume (BV/TV), trabecular number (Tb. N), and trabecular spacing (Tb. Sp) were taken to analyze the mineralized bone tissue. Mice in control group and D-gal-MSC group showed a similar increase trend in Tb.BMD, BV/TV, and Tb. N and decrease trend in Tb.BS/BV and Tb. Sp with statistical difference as compared to the D-gal group (all *p* < 0.05). Similarly, the parameters of Tb.BMD, BV/TV, and Tb. N in D-gal-MSC-TNF*α* group were higher than those in D-gal group (*p* < 0.05), while there was no statistical difference of Tb. Th and Tb.Sp. These data demonstrate sparsely trabecular bone mass after D-galactose treatment, and HUCWJCs reversed the loss of bone mass. Besides, when comparing the trabecular bone parameters between D-gal-MSC and D-gal-MSC-TNF*α* groups, though parameters of Tb.BMD, BV/TV, and Tb. N in D-gal-MSC-TNF *α* group were lower and Tb. Sp and BS/BV were higher than those in D-gal-MSC group, only BS/BV showed statistical significance. This means that TNF*α* combined with HUCWJCs did not have an effective therapeutic effect in the treatment of osteoporosis (Figures 1(c) and 1(d)). Taking the control group as a reference, H&E stains showed the trabecular bone area/total area at distal femur in D-gal group was 0.613 times that of control group (*p* < 0.05), and the ratios in D-gal-MSC (0.944 times of control group) and D-gal-MSC-TNF*α* groups (0.810 times of control group) were higher to that of D-gal group (*p* < 0.05), while there is no statistical difference between the D-gal-MSC and D-gal-MSC-TNF*α* groups (*n* = 3). This further indicated that HUCWJCs rescued the osteoporosis phenotype induced by oxidative stress (Figure 1(e)).

### 3.2. HUCWJCs  Treatment Rescues Osteoblasts Differentiation, Proliferation, and Apoptosis Induced by Oxidative Stress *In Vitro*

**Figure 2:**
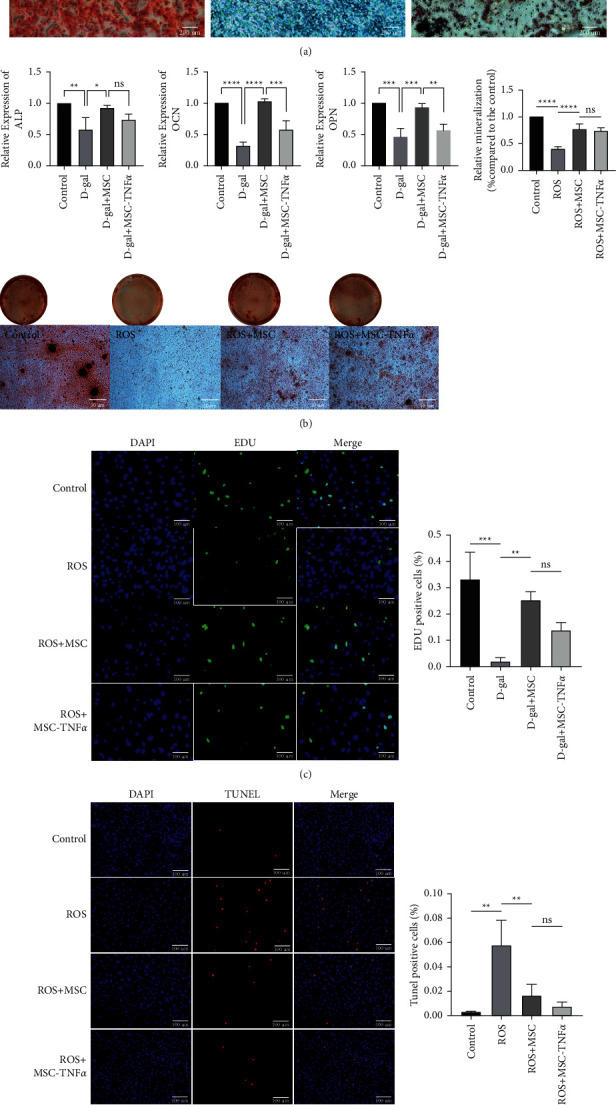
HUCWJCs promoted proliferation and differentiation and inhibited POB induced by oxidative stress in vitro. (a) HUCWJCs differentiated along osteoblasts, lipids, and chondroblasts. (b) Alizarin Red S staining of POB at day 14 of osteogenic induction. (c ∼ d) Proliferation and apoptosis of POB which were evaluated by EDU and TUNEL staining after being treated in different groups. All data is reported as the mean ± SD. Statistical significance was determined by one-way ANOVA. ^*∗*^*p* < 0.05_,_^*∗∗*^*p* < 0.01, ^*∗∗∗*^*p* < 0.001; ns = not statistically significant.

Identification of stem cell pluripotency of HUCWJCs toward osteoblasts, adipocytes, and chondrocytes was confirmed in vitro (Figure 2(a)). The expression of osteogenic mRNA (ALP, OCN, and OPN) was significantly decreased in the ROS group compared to the ROS + MSC and control groups (*p* < 0.05, Figure 2(b)). Besides, the mRNA expression levels of OCN and OPN were significantly lower in ROS + MSC-TNF*α* group when comparing to the ROS + MSC group with statistical difference (*p* < 0.05). POB were then subjected to Alizarin Red S staining at day 14 (60504ES25, YEASEN, China) to identify calcium deposits in the cell layers, followed by quantification. The ROS group showed less osteogenic ability compared to the control group, while the osteogenic ability of ROS + MSC group and ROS + MSC-TNF*α* group was significantly stronger than that of ROS group, and there was no significant difference between ROS + MSC group and ROS + MSC-TNF*α* group (Figure 2(b)). To gain insights into the cellular mechanism of osteogenesis defects caused by oxidative stress, EDU & TUNEL staining were performed to detect proliferation and apoptosis of POB. Interestingly, the results indicated that the proliferation of POB decreased (*p* < 0.05), and the level of apoptosis was upregulated in the ROS group compared to the control group (*p* < 0.05). HUCWJCs treatment promoted proliferation and inhibited POB apoptosis (*p* < 0.05). Meanwhile, there is no statistical difference of proliferation and apoptosis between the ROS + MSC and ROS + MSC-TNF*α* groups (*p* > 0.05, Figure 2(c) and 2(d)).

### 3.3. HUCWJCs Treatment Enhances the Invasion and Migration Ability Induced by Oxidative Stress

**Figure 3:**
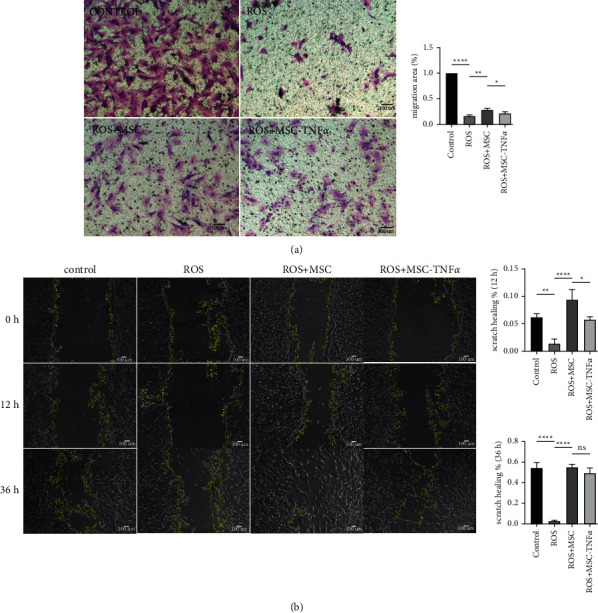
HUCWJCs enhance the invasion and migration ability induced by oxidative stress in vitro. (a) Crystal violet staining of POB from different groups. (b) The migration of POB from different groups. All data is reported as the mean ± SD. Statistical significance was determined by one-way ANOVA. ^*∗*^*p* < 0.05_,_^*∗∗*^*p* < 0.01, ^*∗∗∗*^*p* < 0.001; ns = not statistically significant.

Next, we investigate the effect of HUCWJCs on the invasion and migration ability of POB, which were precultured with or without H_2_O_2_ (400 uM) for 3 h, followed by coculturing with HUCWJCs or HUCWJCs-TNF*α* for different periods of time (0,12,36 h). The wound healing percent in the scratched area in ROS group (0.959 ± 0.275%) is lower than that in ROS + MSC group (10.369 ± 1.118%) and control group (6.454 ± 0.695%) at 36 h (Figure 3(b), *p* < 0.05). There was no statistical difference between control group and ROS + MSC group at 36 h. For invasion assay, with control group as the reference, cells stained by crystal violet were 16.51 ± 2.22% in ROS group. And crystal violet stain area ratio in ROS-MSC group (28.62 ± 2.92%) was higher than that in ROS group (16.51 ± 2.22%) and that in ROS + MSC-TNF*α* group (21.74 ± 3.10%) (Figure 3(a), all *p* < 0.05). This further suggested that HUCWJCs treatment enhances the invasion and migration ability induced by oxidative stress.

### 3.4. HUCWJCs  Activate the Mitogen-Activated Protein Kinase Signaling Pathway Expression of POB

**Figure 4:**
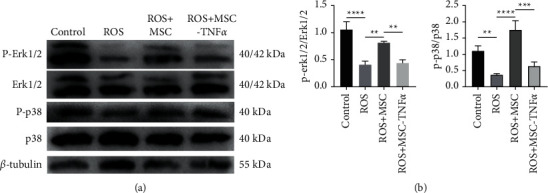
HUCWJCs activate ERK1/2 and p38 mitogen-activated protein kinase signaling pathway expression of POB under oxidative stress. (a) The western blot result of activation of ERK1/2 and p38 MAPK and (b) the quantification of protein expression. All data is reported as the mean ± SD. Statistical significance was determined by one-way ANOVA. ^*∗*^*p* < 0.05, ^*∗∗*^*p* < 0.01, ^*∗∗∗*^*p* < 0.001; ns = not statistically significant.

To investigate the signal transduction system for extracellular signals to intracellular reactions in different groups, protein expression of the mitogen-activated protein kinase pathway was detected. The expression of phosphorylated ERK1/2 to total ERK1/2 in ROS group was 0.388 times that in control group and was 0.504 times that in ROS + MSC group (*p* < 0.05). The expression of phosphorylated p38 to total p38 in ROS group was 0.335 times that in control group and was 0.211 times that in the ROS + MSC group (*p* < 0.05). And ROS + MSC-TNF*α* group had lower expression of phosphorylated ERK1/2 and phosphorylated p38 than that in ROS + MSC group (*p* < 0.05). The data was quantified by ImageJ (Figure 4).

## 4. Discussion

D-galactose has been widely used to establish aging model in mice. According to Qian, chronic D-galactose exposure decreases the activity of catalase and increases the accumulation of lipid peroxidation, advanced glycation end products (AGEs), and malondialdehyde (MDA) [18, 23]. D-galactose has been shown to promote senescence of neural stem cells and neurons through oxidative stress and proapoptotic forms [24]. In our study, D-galactose was used to induce osteoporosis in mice, MDA in serum of D-gal group was significantly higher than that in D-gal + MSC and control groups, and mice in D-gal group showed significantly decreased bone mass when compared to the control group, indicating the oxidative stress-induced osteoporosis model in mice is successful. HUCWJCs have unique and valuable inherent characteristics: first, they can be easily obtained without any risk to the donor and with no ethical controversy; second, HUCWJCs possess low immunogenicity and good antioxidant ability [25–27], which means low concentration of reactive oxygen species and a high concentration of glutathione (key antioxidant). To further determine how oxidative stress and HUCWJCs treatment affect osteogenesis, the trabecular bone mass at the distal femur was assessed in four groups in our study. Parameters of BMD, Tb. N, and BV/TV at distal femur in D-gal-MSC group were significantly higher than those in D-gal group, which suggests that HUCWJCs prevent the progression of osteoporosis. HUCWJCs-derived extracellular vesicles serve as a critical regulator of bone metabolism and represent a potential agent for prevention and treatment of osteoporosis [28]. Zhang et al. reported coculture of osteoblasts and HUCWJCs promoted the proliferation and osteogenic ability of osteoblasts [29, 30]. HUCWJCs-exosomes also promote cell proliferation and protect against oxidative stress-induced cell apoptosis in vitro by activation of ERK1/2 and p38 [31]. We confirmed that HUCWJCs treatment partially promoted proliferation and migration and decreased apoptosis of POB induced by oxidative stress via activating the mitogen-activated protein kinase pathway in vitro.

Epidemiological studies showed that patients under oxidative stress are more prone to osteoporosis [32]. Oxidative stress has been strongly associated with osteoporosis [33, 34]. We identified osteoporosis progresses in the D-gal group. Yet, no study has reported whether the D-galactose-induced osteoporosis mouse model can be treated by HUCWJCs. So, what is the relationship between the ability of HUCWJCs to inhibit oxidative stress and osteoporosis? D-galactose induces oxidative stress via elevated levels of oxide in the blood [35]. This further triggers an inflammatory response in the plasma by increasing proinflammatory cytokines (including TNF*α*) and decreasing anti-inflammatory cytokine [36]. The imbalance between pro-and anti-inflammatory cytokine has been considered a causative factor of diminished bone mineral density in osteoporosis [37]. Also, proliferation and differentiation of osteoblasts could be induced by stimulating ERK, JNK, and p38 [38, 39], while oxidative stress could inhibit the MAPK signaling pathway and induce apoptosis of cells [40, 41]. As reported previously, MSC-derived exosomes inhibit osteoporosis by promoting the proliferation of osteoblasts via the MAPK pathway [42]. Therefore, the suppression of inflammation by HUCWJCs helps prevent the development of osteoporosis by balancing inflammatory factors and maintaining the activity of osteoblast MAPK signaling pathway. Also in our study, we found the proliferation and migration abilities of POB decreased in the ROS group when compared to the other groups, while HUCWJCs treatment partially promoted proliferation and migration and decreased apoptosis of POB induced by oxidative stress by activating the MAPK signaling pathway.

In addition to the antioxidant capacity of HUCWJCs, we believe that there are two main reasons explaining why HUCWJCs inhibit the process of osteoporosis. Firstly, altered differentiation potential of MSCs that favor adipocytes rather than osteoblasts is one main cause for osteoporosis; therefore, a treatment strategy aimed at augmenting endogenous MSCs could be a potential method for osteoporosis therapy [43]. Secondly, in vivo trials suggested that MSC-exosome promotes angiogenesis, which in turn prevents the process of osteoporosis [44]. In addition, to investigate whether HUCWJCs affect the progression of osteoporosis through inflammation, TNF*α*, one of the major inflammatory factors, was included in our study. We hypothesized that coculture of TNF*α* and HUCWJCs would inhibit HUCWJCs' anti-inflammatory and antiosteoporosis effects. However, in the whole experimental process, compared with the D-gal + MSC group, the D-gal-MSC-TNF*α* group showed a certain degree of bone mass reduction in *μ*CT and histology, but there was no statistical difference. Only the expression of osteogenesis related mRNA and the changes of invasion and migration in vitro showed statistical difference. This was not exactly consistent with our expectation, and we believed that there were several probable causes for this result. (1) The time of coculture of HUCWJCs and TNF*α* was insufficient. (2) The in vitro culture of TNF*α* may adversely affect its activity. Besides, this study has several limitations. Firstly, hydrogen peroxide as an oxidative inducer in vitro does not represent the level of oxidative stress induced by D-galactose in vivo completely. Secondly, there is no clear conclusion about the anti-inflammatory and antiosteoporosis effects of TNF*α* on HUCWJCs, which may be related to the above reasons and needs to be further studied.

## 5. Conclusion

HUCWJCs can prevent the progression of osteoporosis by inhibiting oxidative stress, which may act by regulating osteoblasts fate through the MAPK pathway.

## Data Availability

The data that support the findings are available from the corresponding author (Desheng Wu) upon reasonable request.
